# Location of Violent Crime Relative to Trauma Resources in Detroit: Implications for Community Interventions

**DOI:** 10.5811/westjem.2019.9.44264

**Published:** 2020-01-27

**Authors:** Michael J. Clery, Daniel A. Dworkis, Tolulope Sonuyi, Joneigh S. Khaldun, Mahshid Abir

**Affiliations:** * Emory University, Department of Emergency Medicine, Atlanta, Georgia; † Emory University, The Injury Prevention Research Center at Emory, Atlanta, Georgia; ‡ Keck School of Medicine of USC, Department of Emergency Medicine, Los Angeles, California; § The Lever Institute, Los Angeles, California; ¶ University of Michigan, Acute Care Research Unit, Ann Arbor, Michigan; || Wayne State University, Department of Emergency Medicine, Detroit, Michigan; # Michigan Department of Health and Human Services, Lansing, Michigan; ** Henry Ford Hospital, Department of Emergency Medicine, Detroit, Michigan; †† RAND Corporation, Santa Monica, California; ‡‡ University of Michigan, Department of Emergency Medicine, Ann Arbor, Michigan; §§ University of Michigan, Institute for Healthcare Policy and Innovation, Ann Arbor, Michigan

## Abstract

**Introduction:**

Detroit, Michigan, is among the leading United States cities for per-capita homicide and violent crime. Hospital- and community-based intervention programs could decrease the rate of violent-crime related injury but require a detailed understanding of the locations of violence in the community to be most effective.

**Methods:**

We performed a retrospective geospatial analysis of all violent crimes reported within the city of Detroit from 2009–2015 comparing locations of crimes to locations of major hospitals. We calculated distances between violent crimes and trauma centers, and applied summary spatial statistics.

**Results:**

Approximately 1.1 million crimes occurred in Detroit during the study period, including approximately 200,000 violent crimes. The distance between the majority of violent crimes and hospitals was less than five kilometers (3.1 miles). Among violent crimes, the closest hospital was an outlying Level II trauma center 60% of the time.

**Conclusion:**

Violent crimes in Detroit occur throughout the city, often closest to a Level II trauma center. Understanding geospatial components of violence relative to trauma center resources is important for effective implementation of hospital- and community-based interventions and targeted allocation of resources.

## INTRODUCTION

In 2015 there were 1,759.6 violent crimes per 100,000 residents in Detroit, Michigan, the second highest rate in the nation. In 2018 the Federal Bureau of Investigation named Detroit the second most dangerous city in America.^1^ Violence disproportionately affects Black adolescents, for whom homicide is the leading cause of death compared to accidental trauma for the general adolescent population.^2^ Beyond fatalities, the Centers for Disease Control and Prevention reports that for every homicide there are 94 non-fatal violent injuries.^3^ Youth who have been injured are at increased risk for further injury and death, with 44% of injured youth admitted to an urban hospital trauma service later readmitted for assault and 20% ultimately killed within five years.^4^

After treatment at a medical center, victims of violent crime are often discharged back to the same environment in which they were injured, placing them at risk for continued violence and injury.^5^ preventative interventions are often based out of inpatient units; however, the question of how often youth who have been injured due to violence are discharged from emergency departments (EDs However, hospital- and community-based interventions, such as the Safe Streets intervention in Baltimore, have been proven to decrease youths’ risk of violence,.^6^ For these public health interventions to be effectively and efficiently implemented, they must be appropriately targeted. This is particularly important in cities like Detroit that have relatively low population density (only eight census block groups have greater than 15 housing units per acre, the majority having less than five) spread over 139 square miles.^7^ While Detroit has implemented several projects aimed at stemming violence, targeted strategies may improve effectiveness.^8^

Geospatial mapping has previously been used to implement targeted interventions and to manage chronic disease by “hot-spotting” of acute care use.^6^ Hot-spotting describes a “data driven process for the timely identification of extreme patterns in a defined region of the healthcare system.”^9^ Geospatial mapping has also been used to evaluate the geographic distribution of child abuse cases to deliver targeted interventions, as well as to identify communities with high burdens of opioid-related emergency department (ED) visits and hotspots of opioid overdose.^9^

Examination of geospatial data relative to health system resources is a novel approach to inform not only areas of risk but also opportunities for health system-community partnerships. Hospital- and community- based interventions also require well-developed public health infrastructure and partnerships in order to compete for necessary grants and other funding to support such programs.^10^ The objective of this study was to analyze the location of homicides and violent crimes in Detroit in relation to the city’s trauma centers to explore ways in which hospital- and community-based violence intervention programs could be optimally deployed.

**Population Health Research Capsule**

What do we already know about this issue?

There are effective hospital- and community-based intervention programs to reduce violence, but they require significant resources and coordination with trauma systems.

What was the research question?

We examined optimal deployment of intervention resources and show a reproducible process for such evaluation.

What was the major finding of the study?

One trauma center was closest to >40% of violent crime in Detroit; most violent crimes occurred closest to a Level II trauma center.

How does this improve population health?

Examination of violent crimes or other public health issues relative to health center resources can inform optimal intervention deployment.

## METHODS

We obtained data on the type and location of all crimes in Detroit from 2009–2015 from a publicly available database maintained by the Detroit Police Department via the Detroit Open Data Portal. Crime locations are blurred slightly by the addition of a small amount of spatial “noise” to ensure anonymity but remain accurate to the street block level. Crimes were tagged as “violent” if they were identified as one of the following categories: “AGGRAVATED ASSAULT,” “ASSAULT,” “HOMICIDE,” “JUSTIFIABLE HOMICIDE,” or “NEGLIGENT HOMICIDE.”

Data on the locations of hospitals in Detroit were obtained from “Data Driven Detroit,” a publicly available database. Since the Department of Veterans Affairs Detroit Medical Center, Detroit Receiving Hospital, Hutzel Hospital, Harper Hospital, and Children’s Hospital of Michigan are all located in the same hospital complex in downtown Detroit, we created a composite “Downtown Medical Center (DTMC)” surrogate, with coordinates defined as the unweighted geometric average of these hospitals. We performed centroid analysis using the “geosphere” package executed in the R programming language (www.r-project.org). Distances between crimes and hospitals were calculated using the Vincenty ellipsoid method, executed via the “geosphere” package with an equatorial axis of 6,378,137 meters (m), a polar axis of 6,356,752.3142 m, and an inverse flattening of 1/298.257223563. For each violent crime we identified the closest hospital within the city and the distance to that hospital. All further statistical analyses were performed in the R language. This study was determined to not require review by the Emory Institutional Review Board.

## RESULTS

During the study period, 1,083,265 crimes were recorded by the Detroit Police Department, including 202,931 violent crimes (18.7%). Table 1 shows the breakdown of crimes by year. While overall numbers of crimes decreased from approximately 181,000 per year to approximately 137,000 per year, the percent of those crimes that were violent rose from 17.6% to 20.5%.

The median distance between a crime and the closest available hospital was 4.582 kilometers (km) (inter-quartile range [IQR] 2.682 km – 6.428 km). Violent crimes were slightly, although significantly, farther away from hospitals than nonviolent crimes (median distance 4.7 km with IQR 2.9 km – 6. 5km compared to median distance 4.5 km with IQR 2.6 km – 6.4 km, p-value < 2.2E-16). Among the subset of 202,931 violent crimes, 200,348 (98.7%) occurred within 10 km (6.2 miles) of a hospital, 108,918 (53.7%) occurred within 5 km, and 8,782 (4.3%) occurred within one km. Across the study period, the median distance between a violent crime and the closest hospital stratified by year of crime varied minimally.

Over the study period, the hospital closest to violent crimes was most often Sinai-Grace Hospital, which was the closest hospital for 41.2% of violent crimes followed by Henry Ford Hospital (23.6%), Ascension St. John Hospital (19.2%), and DTMC (16.0%). Table 2 depicts the breakdown of violent crimes by closest hospital across each year of the analysis period and shows that these trends are stable across the analysis period. Of the four hospital complexes considered here, the DTMC and Henry Ford hospitals carry Level I trauma designations, while St. John’s and Sinai-Grace are Level II trauma facilities. Among violent crimes, only 39.5% occurred closest to a Level I trauma center, while the majority (60.5%) occurred closest to Level II trauma centers.

## DISCUSSION

We explored the spatial relationship between violent crimes and trauma centers in Detroit and showed that the majority of violent crimes occur close to hospitals, within five km in most cases. These findings are especially relevant in a city like Detroit with low population density. This analysis aims to understand the best approach to administer resources for violence prevention and intervention in relation to a hospital partner, not simply relative to crime location. Based on this geospatial analysis, Sinai Grace Hospital and the surrounding communities would likely realize the greatest benefit from investment in community violence reduction interventions.

Presenting information with geospatial data centered around well-known community landmarks has previously been employed to engage community organizations and empower community members to organize effectively. The ability to organize multiple stakeholders around an issue would likely be important for violence, which disproportionately affects a marginalized population for whom social support is often lacking.^11^ In prior work, researchers reported: “The repeated display of health-disparity hot spot maps ensured that multiple audiences could quickly interpret prevalence and trends.”^12^ This analysis of violent crime relative to a well-known trauma center location could also be used as a model for community engagement around effective investment in hospital- and community-based interventions for many public health issues.

While resources for trauma care are, by design, concentrated at Level I trauma centers, the communities suffering from violent crimes in Detroit are more often closer to Level II trauma centers. A single Level II trauma center was the closest hospital to over 40% of all violent crimes in Detroit. Based on these results, policymakers and payers should consider incentivizing Level II trauma centers to prioritize violence-related prevention and interventions to optimally address the safety and well-being of the communities they serve. Although they are not necessarily where every patient in the area is treated for injuries, these centers represent opportunities for community-based health system partnership to reduce injury. Further, nonprofit hospitals with Level II trauma centers should consider supporting violent injury prevention as a key strategy to meet community benefit requirements for federal tax exemption.

## LIMITATIONS

This study has several limitations. First, the analysis relies on a single data source, the Detroit Police Department, with the likely result that not all violent crimes actually occurring in Detroit were included in the analysis. A recent study indicates a large difference in the number of violent injuries reported to police and treated in a trauma center, further reflecting the necessity of health system involvement.^13^ Second, we only included hospitals within the city of Detroit, likely resulting in under-sampling the places in which victims of violent crime go to receive care. We also did not consider transportation times or routes that may affect the hospital receiving an injury. This study focused on violent crime and proximity to hospitals in one urban city and is not necessarily generalizable to other localities.

## CONCLUSION

The burden of violent injuries in Detroit requires using geospatial data to focus health system efforts of harm reduction and prevention. Evaluating the spatial relationships between violent crimes and trauma centers can serve as a critical tool for strategic assessment of communities at most risk, informing both resource allocation and key partnerships between the community and healthcare systems. Further, this work can inform the research agenda and policy around violent crime prevention and intervention implementation in order to improve the life and health of Detroit residents and other urban centers using a similar approach.

## Figures and Tables

**Table 1:** Comparison of the numbers of total, violent, and nonviolent crimes reported by the Detroit Police Department from 2009 to 2015.

Year	Total crimes	Nonviolent crimes	Percent	Violent Crimes	Percent
2009	181,427	149,549	82.4%	31,878	17.6%
2010	169,925	138,961	81.8%	30,964	18.2%
2011	156,569	128,172	81.9%	28,397	18.1%
2012	155,581	126,831	81.5%	28,750	18.5%
2013	146,679	119,447	81.4%	27,232	18.6%
2014	136,359	108,672	79.4%	27,687	20.3%
2015	136,725	108,702	79.5%	28,023	20.5%

**Table 2:** Counts of which violent crimes are closest to each hospital, broken down by year of analysis.

	2009	2010	2011	2012	2013	2014	2015
DTMC	5,399	5,188	4,792	4,673	4,189	3,999	4,072
Sinai-Grace	12,529	12,266	11,222	11,810	11,581	12,014	12,345
Henry Ford	7,925	7,457	6,900	6,738	6,394	6,207	6,315
St. John	6,025	6,053	5,483	5,529	5,068	5,467	5,291

*DTMC*, Downtown Medical Center.

Level I trauma centers: DTMC, Henry Ford; Level II centers: Sinai-Grace, St John’s.
